# The combined effect of a carbon sleeve and carbon veil as an anode electrode in enhancing microbial fuel cell performance

**DOI:** 10.1039/d5ra05180k

**Published:** 2025-11-03

**Authors:** Aradhana Singh, Yi Wang, Ioannis A. Ieropoulos

**Affiliations:** a Water & Environmental Engineering Group, School of Engineering, University of Southampton, Bolderwood Campus SO16 7QF UK i.ieropoulos@soton.ac.uk

## Abstract

In this study, a novel composite anode electrode for microbial fuel cells (MFCs) was developed by wrapping a carbon sleeve around the surface of carbon veil. While the carbon veil is widely used due to its biocompatibility and surface area, its poor mechanical strength limits long-term operation. The novelty of the study lies in reinforcing the flexible, non-woven carbon veil with a mechanically robust and tubular carbon sleeve layer without compromising performance. This composite electrode (carbon veil + carbon sleeve) improved mechanical integrity and in addition, facilitated better biofilm development. Over a 5 week operation, the composite anode achieved a maximum power output of 527.7 μW, a 3-fold increase compared to using the carbon veil alone (175.5 μW). SEM analysis confirms biofilm improvement in the combined electrodes compared to the control. FTIR analysis confirmed changes in surface functional groups post-operation, suggesting enhanced biofilm–electrode interactions. This work demonstrates a simple yet effective reinforcement strategy that significantly enhances MFC anode durability and power performance.

## Abstract 2

In this study, a novel composite anode electrode for microbial fuel cells (MFCs) was developed by wrapping a carbon sleeve around the surface of a carbon veil.

## Graphical abstract

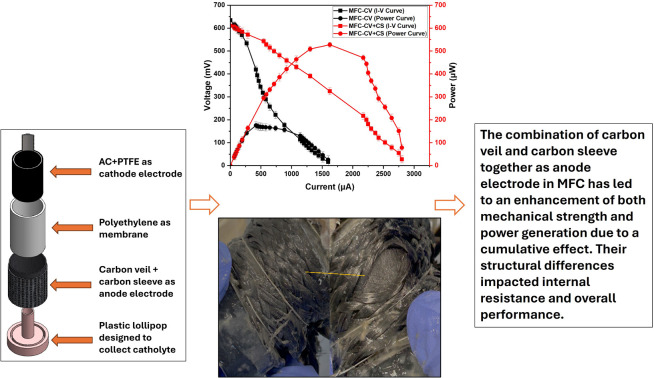

## 1 Introduction

The microbial fuel cell (MFC) has emerged as a technology that harnesses the potential of microbes to treat wastewater and generate electricity simultaneously. It is particularly effective in charging low-power applications such as mobile phones, wall clocks, sensors, and a range of other devices.^1,2^ A key factor influencing MFC performance is the quality and functionality of the anode electrode, which directly affects biofilm formation, electron transfer, and overall performance output. There are many criteria, such as conductivity, porosity, inertness, mechanical strength, and biocompatibility, important to support good working of a high-performance MFC.^3,4^ Unlike conventional fuel cells, MFC anodes must support microbial biofilm formation to facilitate electron transfer from microbes to the electrode surface. Carbon-based materials have emerged as strong candidates due to their good electrical conductivity, large surface area, chemical inertness, and ability to support microbial growth.^5,6^ In previous studies, commercial carbon materials such as carbon paper, felts, cloths, graphite, nanotubes, graphene, and others are widely applied as electrode materials in MFCs.^7,8^

Carbon veils, in particular, have been widely used due to the above mentioned qualities and in addition, low-cost and easy handling.^9,10^ Ieropoulos *et al.* conducted experiments in 2008 that demonstrated the effectiveness of a carbon veil as an anode electrode in MFC, making it ideal for scaling up by stacking numerous individual MFCs.^9^ One of the advantages of using a carbon veil is that it can be conformed to fit into any shape, while leaving enough surface area for biofilm formation.^11^ Despite the many benefits of employing carbon veil, there are two shortcomings associated with it that need to be addressed. First, because of its thin fabric, it has low tensile strength, and it can easily rupture when assembled MFC modules are moved. Secondly, compared to charcoal or biochar, it has a lower surface area, but higher conductivity, and compared to catalysts *e.g.*, platinum or carbazine, the carbon veil underperforms in terms of rates of reaction, but is a lot less costly. Therefore, strategies should be developed to enhance its mechanical properties, increase its durability, and improve bacterial colonisation. Previous reinforcement strategies such as coating carbon veils with activated carbon ink^12^ have improved conductivity and porosity but have not adequately addressed mechanical durability or long-term performance under operational stresses.

This study introduces a novel reinforcement strategy that addresses both the aforementioned weaknesses. By combining the high mechanical strength and flexibility of a woven carbon sleeve, a material commonly used in aerospace and medical applications,^13–15^ with the advantages of carbon veils, this study proposes a composite anode that enhances durability, preserves biocompatibility, and potentially improves biofilm formation. Unlike prior experiments that modify the carbon veil surface or embed catalysts, this approach employs structural reinforcement to improve electrode integrity over extended operation and optimise performance under variable feeding conditions. The proposed carbon sleeve and carbon veil also exploit the conformability of both materials. The carbon sleeve was shown to be a high-performing electrode during the testing of tubular-shaped MFCs.^16^ This finding was attributable to the carbon sleeve's tubular design, which made it considerably more advantageous for tubular-shaped MFCs.

In this study, we evaluate the performance of this novel composite carbon sleeve reinforced carbon veil anode over a 5 month operational period, under both continuous and starvation feeding regimes. We compare its performance to that of the traditional approach of using carbon veil electrodes through polarisation experiments at different time intervals. Surface morphology and characterisation *via* SEM and FTIR spectroscopy provide insights into the changes in microbial dominance and functional groups of the materials. This work closes a critical technical gap by addressing the mechanical fragility of carbon veil anodes without sacrificing their electrochemical performance, offering a scalable, cost-effective electrode design for long-term MFC deployment.

## 2 Materials and methods

### 2.1 Anode electrode

The anode electrode was used in two different ways in the present study. In the first type, MFC-CV, the carbon fibre veil with a carbon loading of 20 g m^−2^ and a total macro-surface area of 20 × 20 cm^2^ was used as the anode. In the second type of MFC-CV + CS, a carbon veil (20 × 10 cm^2^) was put in the tubular-shaped carbon sleeve of total area (20 × 10 cm^2^). This way, the overall macrosurface area of this MFC-CV + CS anode was kept the same as that of MFC-CV, *i.e.*, 400 cm^2^. Fig. S1 illustrates how the carbon veil was put inside the carbon sleeve. In this process, the carbon veil, a lightweight, nonwoven carbon fibre fabric, was carefully rolled or folded to match the diameter and length of the carbon sleeve. The veil was then inserted longitudinally into the hollow interior of the carbon sleeve, ensuring even distribution along its length. This setup was designed to ensure close contact between the veil and the inner walls of the sleeve, promoting uniform conductivity and structural integration.

### 2.2 MFC fabrication and inoculation

**Fig. 1:**
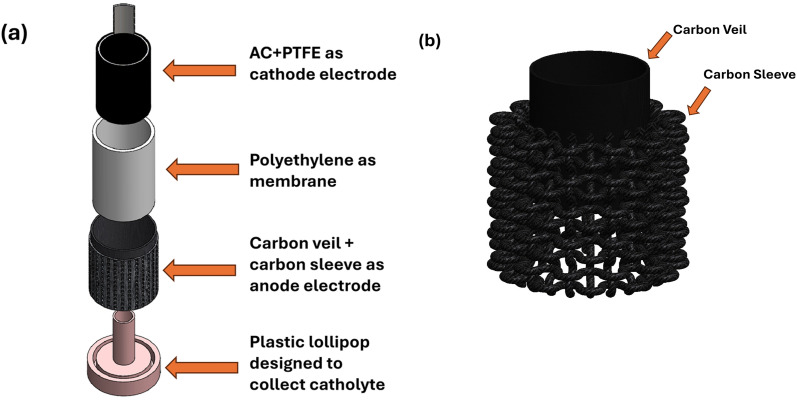
(a) Layout of all the components of MFC used in this study (b) combination of carbon sleeve and carbon veil used together as one electrode.

MFCs with an outer anode and an inner cathode, made of plastic (polyethylene) cylinders with a 15% open porosity were employed as the membrane and chassis, supplied by Siam Cement Group (SCG). The cylinders were *ca.* 3 mm thick, 7 cm tall, and 2.7 cm in diameter. A plastic-based container was used to hold a 175 mL working anode volume. Carbon fibre veil (as described above) was used as the anode for MFC-CV, wrapped around the outside of the cylinder. In MFC-CV + CS, carbon veil was put in the tubular-shaped carbon sleeve. The cathode electrodes were made up of a 60% polytetrafluoroethylene (PTFE) solution (Sigma-Aldrich) and activated carbon (G. Baldwin & Co.) distributed over a stainless-steel mesh support. The cathode was prepared using the procedure reported by Gajda *et al.*^17^ The cathode electrodes were inserted into the interior of the plastic cylinders after being cut with a geometric area of 5 × 6 cm^2^ and then manually inserted into the cylinders, held open to the air, and pressed against the wall for improved contact. Both cathode and anode connections were made using Ni-Cr wire. Subsequently, in equal volumes, assembled MFCs were inoculated with a 1 : 1 mixture of raw activated sludge and substrate [1% tryptone, 0.5% yeast extract, and 20 mM sodium acetate]. To prevent the biofilm from starving, 40 mL of the anolyte was taken out each day and replaced with 40 mL of 1% tryptone, 0.5% yeast extract, and 20 mM sodium acetate. Experiments were carried out with triplicate MFCs. The layout of all the components of the MFC used in this study is shown in Fig. 1.

### 2.3 Electrochemical characterisation

The closed-circuit voltage of the MFC [CV] and MFC [CV + CS] was continuously recorded using a Pico technology (ADC-24) data logger. The resistors were gradually reduced in value from 2700 Ω to 390 Ω following a biofilm maturation strategy. The current and power output were calculated based on Ohm's law *i.e. I* = *V*/*R*, where *V* is the voltage in a closed circuit and *R* is the resistance value and *I* is the current. Power was calculated as *P* = *V*× *I*. Polarisation experiments were carried out by varying the resistance from 10 000 Ω to 10 Ω every 3 minutes using a decade resistance box (ELC, DR06). Maximum power was determined from the *P*–*I* graph.

### 2.4 Morphological and spectroscopic characterization of electrodes

Field Emission Scanning Electron Microscopy (FE-SEM) using a JEOL JSM 6500F was performed to examine the biofilm distribution on the electrodes after operation in MFCs. Fourier transform infrared spectroscopy (FTIR) (VHX) was performed on the electrodes to observe the functional group variability before and after the operation of the MFCs.

## 3 Results

### 3.1 Effect of the composite electrode on the biofilm after operation

**Fig. 2:**
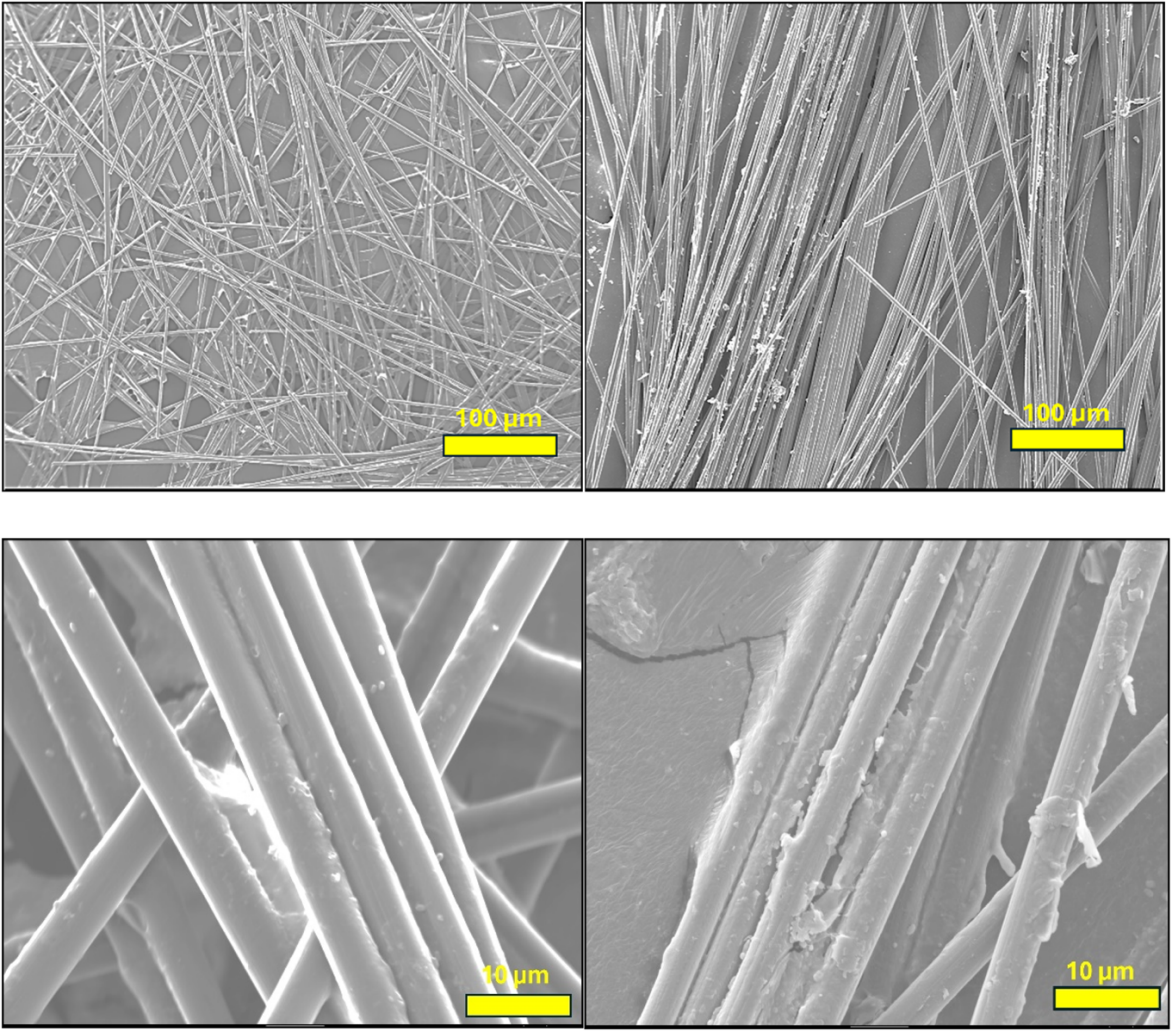
SEM images of electrodes after operation in MFCs at two different magnifications. Left: carbon veil as a single electrode; right: carbon sleeve in combination with carbon veil.

SEM analysis was performed to analyse the biofilm dominance on the electrodes over the carbon sleeve wrapped over carbon veil and the single carbon veil electrode after operation of MFCs for five months. Fig. 2 shows SEM images of the surface morphology of carbon veil used as a standalone anode (left) and the carbon sleeve when integrated in combination with the carbon veil (right). The standalone carbon veil electrode shows relatively less dominance and clean fibre surfaces, indicating limited biofilm development. This could be attributed to the inherently lower surface area utilisation or disintegration of biofilm due to the mechanical failure of the tensile strength of the carbon veil over time. This suggests that the carbon veil creates less favourable structural features alone for microbial attachment and long-term retention. In contrast, the SEM image of the carbon sleeve used in combination with carbon veil, indicates a more irregular surface morphology with more microbial deposits and extracellular polymeric substances (EPS) accumulation along the fibres. The more pronounced roughness and residual deposition suggest improved microbial attachment and biofilm development, contributing to enhanced electrochemical performance. These morphological differences support the hypothesis that the combination of carbon sleeve and carbon veil provides a synergistic effect, enhancing both microbial colonisation and electron transfer efficiency. The increased surface complexity and better conductive network likely facilitate more robust and stable microbial-electrode interactions over long operational periods.

### 3.2 Start-up voltage

**Fig. 3:**
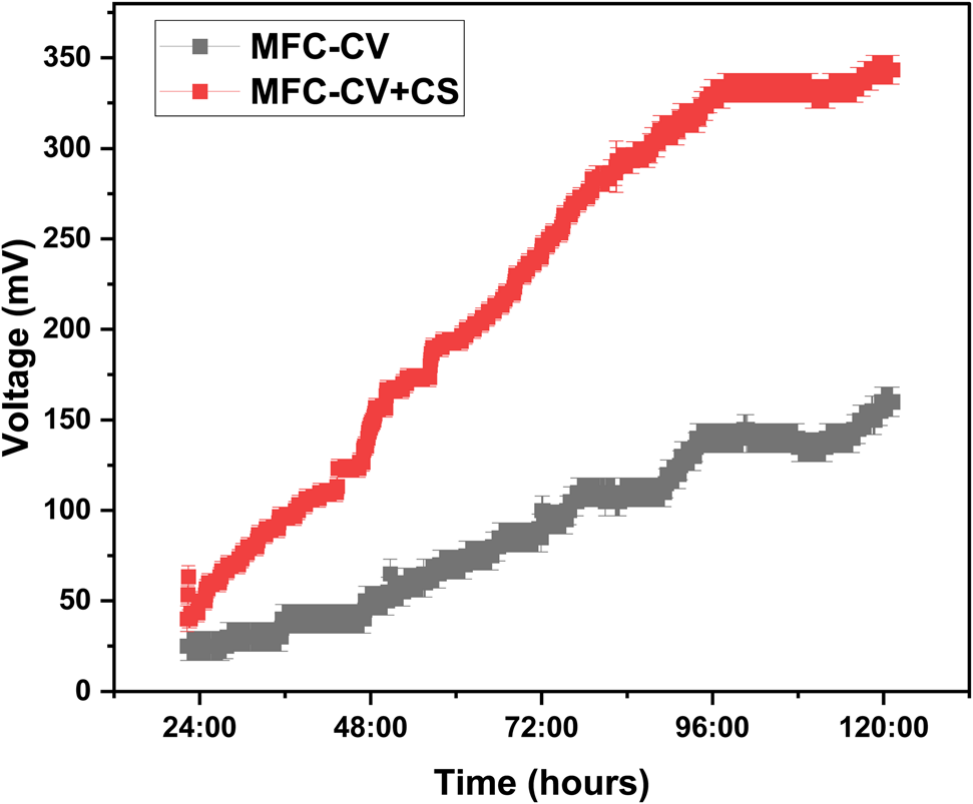
Voltage production over time at 2.7 kΩ in MFC-CV and MFC-CV + CS. Tests were run in triplicate.

Electrical output in MFCs was investigated for the combination (carbon veil + carbon sleeve) electrode and the single electrode (carbon veil). After operating in OCV for 6 hours, a starting load of 2.7 kΩ was added to each MFC (Fig. 3). The findings unequivocally demonstrated that the combination of carbon veil and carbon sleeve had aided in shortening the time needed for microbial attachment to begin; in all three MFC triplicates, a very noticeable increase in voltage was observed when the carbon veil and carbon sleeve combination was employed as the anode electrode.

The carbon sleeve and carbon veil were combined in such a way that the carbon sleeve had enough macropores to expose the carbon veil's surfaces. The combined benefits of both electrodes can be exploited in this way. Start-up time for voltage generation in MFC is strongly influenced by how quickly a stable and efficient microbial biofilm forms on the anode.^18^ The exposed porosity of the electrodes and substrate uptake by the microbes on the electrode are two critical parameters that affect the start-up voltage in MFCs. Prior research has already described the advantages of using carbon veil as an anode electrode.^9,10^ There is not much research done on using a carbon sleeve as an anode electrode or as an electrode reinforcement technique, particularly in MFC-based research.

Fig. S2 is also included in the supplementary document, from a different experimental setup, where the objective was to compare individual electrodes' performance, *i.e.*, MFC-CV *vs.* MFC-CS. A preliminary experiment was conducted for 17 days, employing two types of MFCs (MFC-CV, where carbon veil was the standalone electrode, and MFC-CS, where carbon sleeve was the standalone electrode), each ran in triplicate. It was observed that the triplicates of standalone MFC-CS were not performing at the level of MFC-CV. It was hypothesised that this could be due to large void spaces when carbon sleeve is put on the membrane, so it is possible that not all of the active area of the membrane is covered by the electrode, which results in suboptimal performance. Whereas, when carbon sleeve is used with carbon veil, the combination outperforms the two standalone electrodes. This is because carbon sleeve acts as a reinforcement to the carbon veil and at the same time contributes positively to electron transfer. The optimisation conditions were different, and therefore the results shown in Fig. S2 are not in conformance with this study, but rather to compare the old electrode (carbon veil) with that of the new electrode (carbon sleeve).

### 3.3 Electricity output under different scenarios

#### 3.3.1 Under non-optimal load

**Fig. 4:**
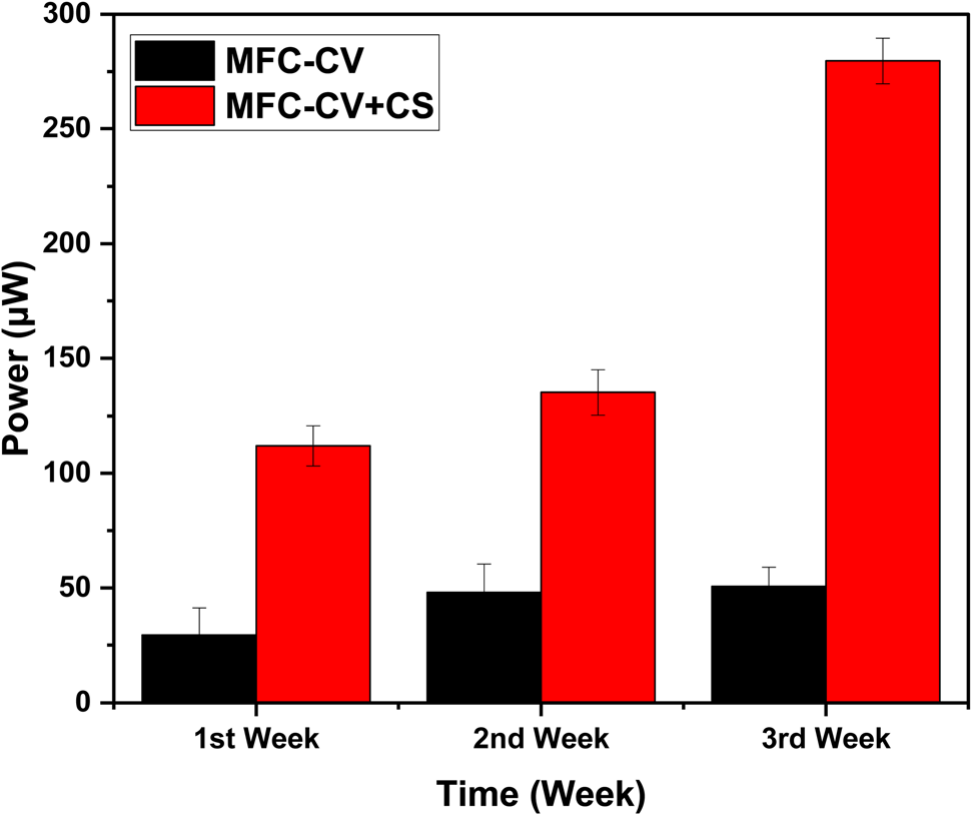
Power output from triplicate MFC-CV and MFC-CV + CS sets over 3 weeks under non-optimized load.

Following inoculation with the substrate and activated sludge, the MFCs were run for a total period of 5–6 months, during which time they were fed constantly for 3–3.5 months. Following this, they were starved for approximately forty days before being fed again. Initially, the comparison was made on the starting (sub-optimal) load in both kinds of MFCs. The external resistance load value gradually decreased from 2.7 kΩ to 1.5 kΩ in the second week, and subsequently, a 1 kΩ was connected in the third week. For the initial two weeks, the power continuously increased in all the MFCs, although MFC-CV + CS consistently produced higher power output. By the end of the second week, the maximum power achieved (135.17 μW) was 2.85 times higher than that of MFC-CV (48.02 μW). Following the third week of operation, the power in MFC-CV (50.65 μW) began to show a very small increase, whereas the power from MFC-CV + CS (279.6 μW) achieved significant improvement. Fig. 4 represents the power output from these MFCs for the initial period of 3 weeks.

**Fig. 5:**
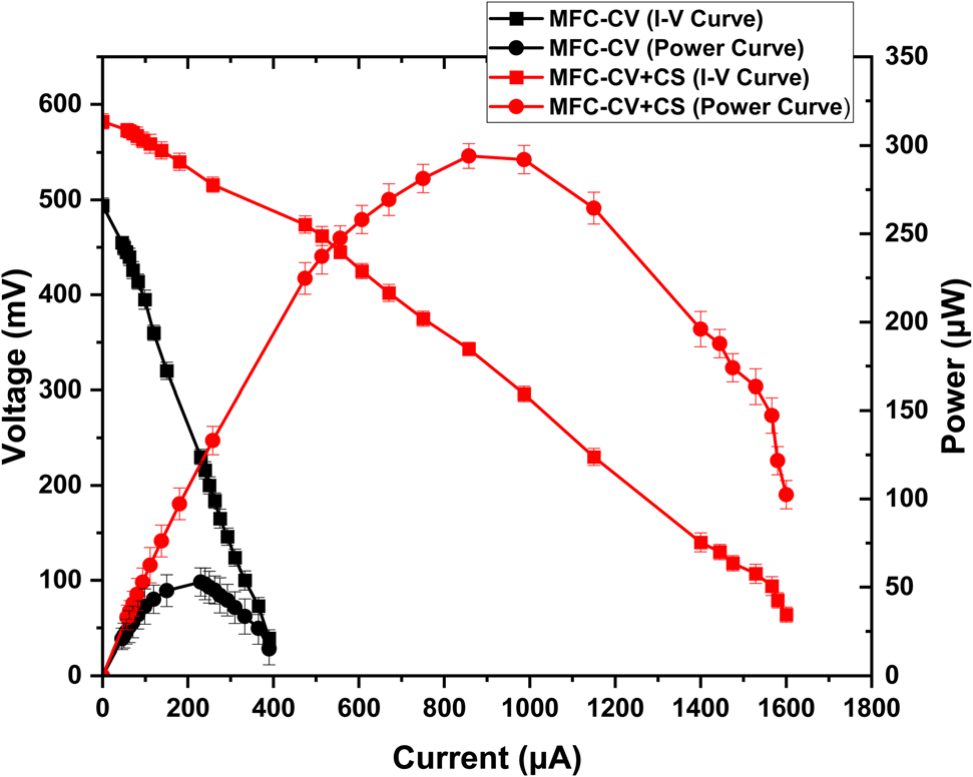
Polarisation carried out at the 3rd week of operation in MFC-CV and MFC-CV + CS.

Polarisation experiments were also carried out during the third week (Fig. 5), which indicated the internal resistance for MFC-CV to be 1 kΩ, producing a maximum power output of 50.2 μW and for MFC-CV + CS to be 400 Ω, producing a maximum power output of 294.13 μW. MFC-CV was therefore operated with the respective optimum load of 1 k, so it is unclear why its performance was continuing to deteriorate. The macro-surface area of both kinds of MFC was the same; however, the resistance seems to be higher in the MFCs that operated with the carbon veil electrode alone. The lower internal resistance found in MFC-CV + CS suggests that the two electrodes coming together in a sort of a parallel ‘circuit’ connection, whereby the total material resistance would tend towards the lowest common denominator. This is perhaps how the difference in internal resistance can be explained which has possibly helped in reducing the different types of loss,^19^ as can be seen in Fig. 5. Particularly, this seems to be more marked for anodic mass transfer limitations. In MFCs, mass transfer limitation on the anode side refers to the restriction in the movement of substrates from the bulk solution to the anode electrode. In a different study, adding an extra water layer over the anode electrode facilitated the mass transfer, and an increased organics flux was observed that resulted in improved power in a sediment MFC.^20^ It seems that the one electrode inside the other acted as a way of creating parallel circuits within the electrode structure, which would have resulted in an overall lower material resistance, in addition to offering enhanced sites for bacterial colonisation.

This was also evident when a preliminary absorption test of substrate on an individual carbon veil electrode and a combination electrode (carbon veil and carbon sleeve) with a similar surface area was carried out (Fig. S3). As can be seen in the SI, the combination electrode facilitated higher absorption of the feedstock than the individual carbon veil electrode.

#### 3.3.2 Under optimum load

**Fig. 6:**
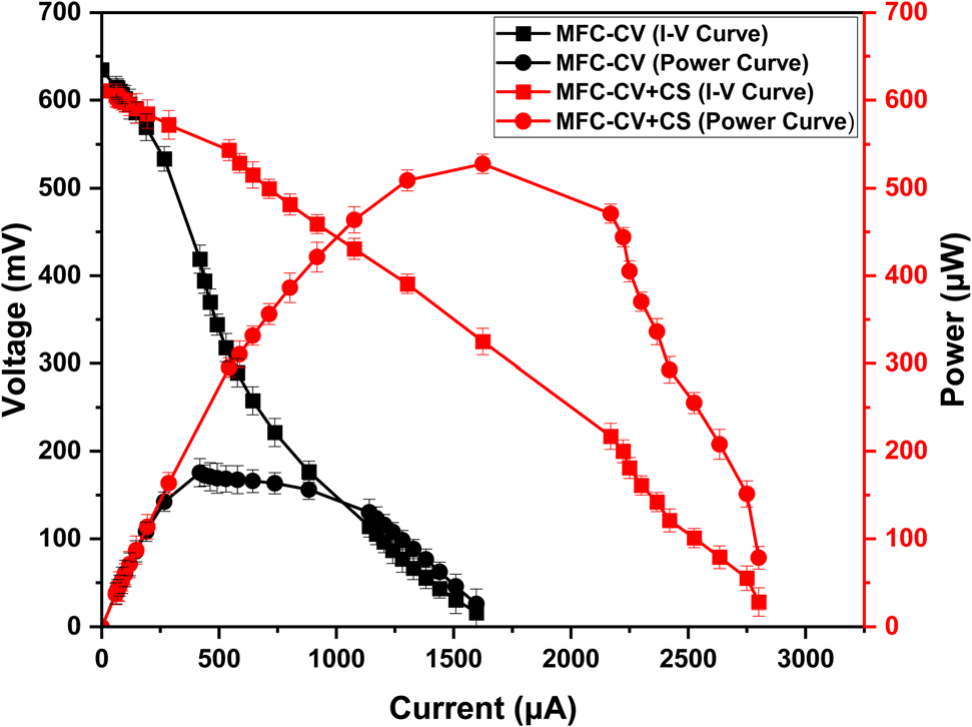
Polarisation carried out at the 5th week of operation in MFC-CV and MFC-CV + CS.

When the optimum load was determined following polarisation in the third week, the load was then adjusted in both types of MFCs. The resistive load was now increased in MFC-CV from 1000 ohms to 1500 ohms (although 1000 ohms was the optimum load for maximum power transfer at this time, the fact that there was no power increase suggests overloading). Whereas the resistive load in MFC-CV + CS decreased from 1000 ohms to 400 ohms. A polarisation was then performed during the fifth week (Fig. 6). By this week, the power in both types of MFCs had increased further to 527.7 μW in MFC-CV + CS, *i.e.*, four times higher than that of MFC-CV (175.46 μW). This was the time, the internal resistance in MFC-CV and MFC-CV + CS was 1000 Ω and 200 Ω, respectively. After 2 weeks of operation, one more polarisation run was performed, which is when the internal resistance in both the MFCs was found to be 100 Ω. However, for the sake of consistency with the next set of experiments, both the MFCs were left at a slightly higher resistance load value, *i.e.*, 390 Ω. It is important to note that under different test conditions, with different/sub-optimal electrode configuration, but optimum load conditions, the same plastic membrane-based MFCs generated 1.5 mW of power over a long period of time (*paper in preparation*).

#### 3.3.3 Regular batch feeding *vs.* starvation

**Fig. 7:**
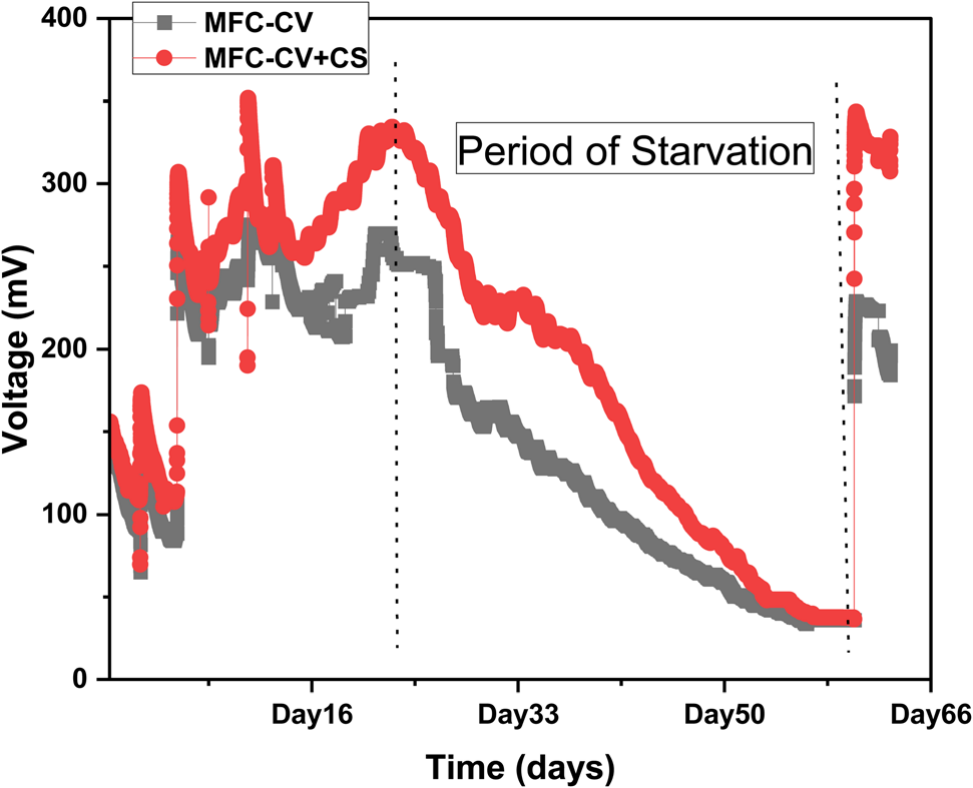
Voltage generation during regular feeding and starvation in MFC-CV and MFC-CV + CS over 2 months.

In real implementation scenarios operated in environments outside the lab, regular feeding cannot always be guaranteed. Another objective of the study was therefore to determine how well these MFCs perform when starved *versus* when regularly fed. The voltage output from MFCs operating around the third month of operation (feeding every third day) and the subsequent 40 days of starvation, followed by the subsequent 10 days of refeeding, is shown in Fig. 7. Before starvation, the average voltage output in MFC-CV + CS (300–350 mV) was higher compared to that given by MFC-CV (258–280 mV) shown in the 12 days (data from the 3rd month of operation when MFC-CV and MFC-CV + CS were operating at 390 Ω with continuous feeding). The MFCs were then starved completely for the next forty days, which caused the voltage to drop (prominent in both types of MFCs). However, after the starvation period, the voltage output quickly recovered in MFC-CV + CS within one day when 80 mL of feedstock was fed. MFCs were run on the same external resistance load that they were running before (390 ohms). MFC-CV + CS reached the equivalent level of performance as before starvation (voltage of 300–350 mV), whereas MFC-CV reached a lower level of performance than before (190–230 mV).

This suggests that the combination electrode outperformed the individual components and allowed the biofilm to respond better to adverse conditions such as starvation. Several studies have investigated starvation recovery in MFCs, including the work by Saheb-Alam *et al.*, which examined the effects of different electron donors on the recovery process following starvation.^21^ Ruiz *et al.* studied the performance of MFCs and microbial electrolysis cells (MECs) under various starvation periods.^22^ It was found that both systems could withstand starvation periods up to 10–11 days without significant performance loss when endogenous consumption was enabled (*i.e.*, closed-circuit operation for MFCs). In the current study, it was observed that the combined electrode played an important role in the health of the biofilm, following starvation.

### 3.4 Electricity production towards the end of the experiments

**Fig. 8:**
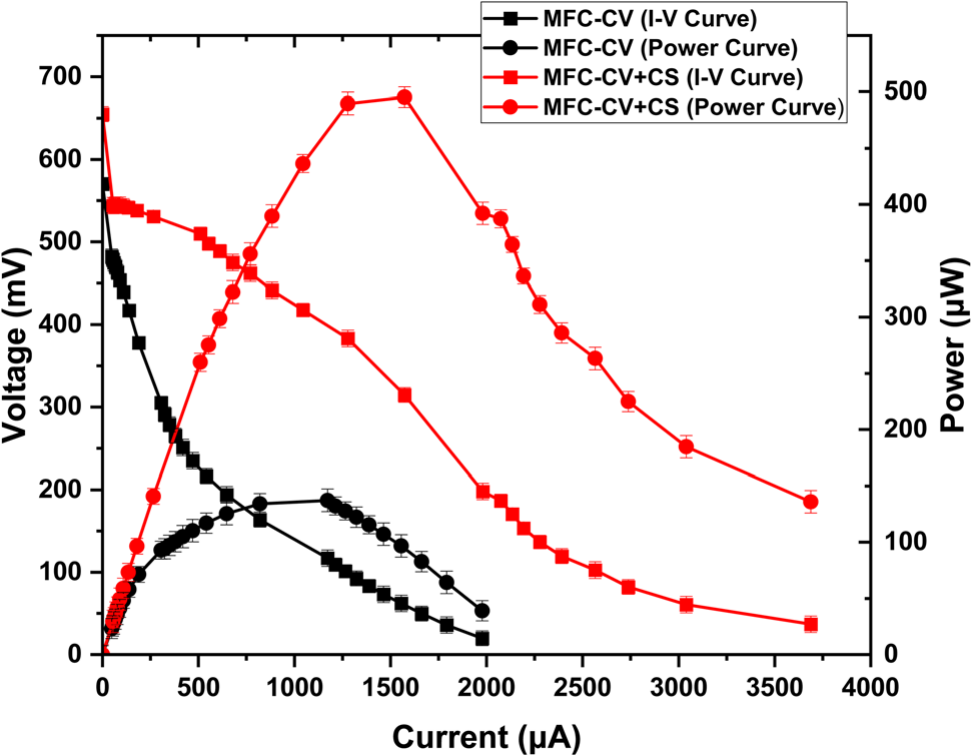
Polarisation run carried out towards the end of the experiment (5th month).

A polarisation experiment (Fig. 8) was carried out by the end of the 5th month to estimate the current and power generation and internal resistance values in these MFCs. The maximum power output of 495.2 μW achieved in MFC-CV + CS was 3.6 times higher than that achieved in MFC-CV (137.3 μW). This shows that when the carbon veil was wrapped by a carbon sleeve, this led to higher microbial interaction of the anode with the substrate, resulting in higher metabolism of microbes to release electrons. This is particularly interesting as it offers a new direction for research in the field. Conventionally, it has been assumed that a higher surface area of the carbon electrode directly correlates with increased power output in MFCs. However, in this case, even though the surface area and type of carbon electrode remained constant, the biofilm integrity was a more critical factor. Carbon sleeve appears to enhance microbial retention, likely by providing a more favourable microenvironment or improved physical entrapment for the biofilm, thereby supporting sustained electroactive activity and stability over time. The high porosity of the carbon sleeve allowed a more robust microbial colonisation on the textured surfaces, enhancing bacterial adhesion and retention. A higher surface area provides more sites for extracellular electron transfer (EET), which is critical for power generation. A well-designed sleeve structure supports efficient diffusion of substrates (like organic matter) into the biofilm. The sleeve's structure also affects its mechanical strength, which is important for long-term stability in real-world applications. In other words, the additional attachment sites provide more material available for colonisation from electroactive bacteria, which immediately increases the charge transfer potential. Synergistic effects between attachment sites of the two electrode materials will need to be carefully studied, quite possibly with optical imaging techniques to assess biofilm biomass *vs.* available electrode material and possibly even quorum sensing investigations, on how microbes on the two different electrodes interact.

## 4 Variation of functional groups on electrodes from these MFCs

**Fig. 9:**
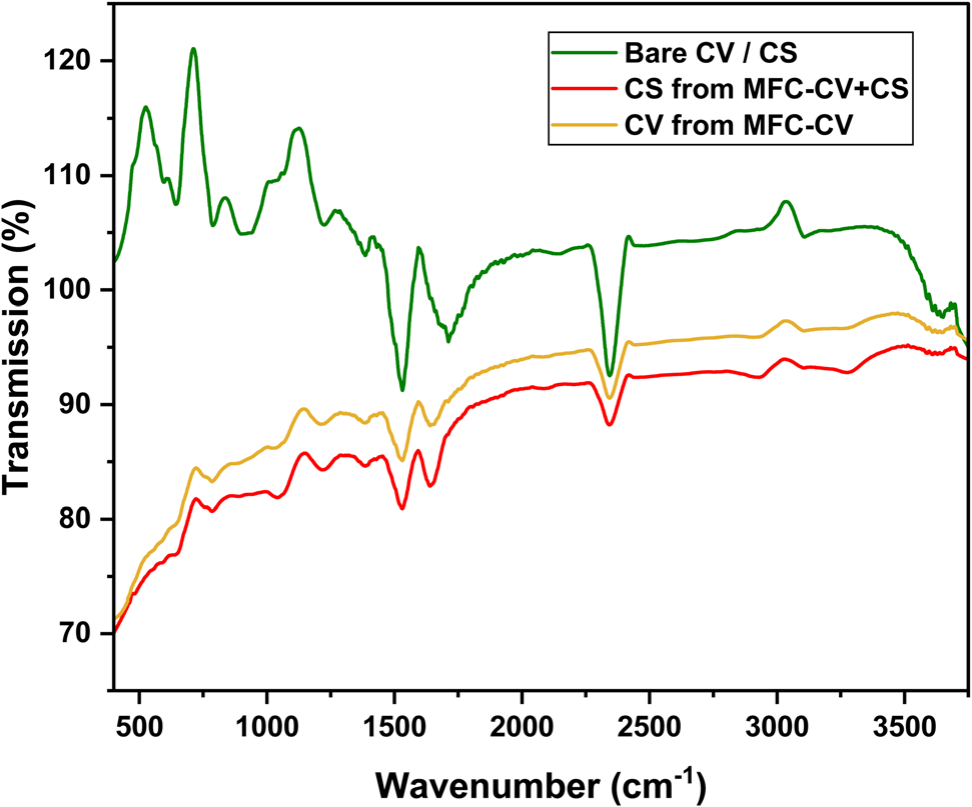
FTIR spectra of the carbon veil electrode (bare, before experiments) and the electrode after operation in MFC-CV and MFC-CV + CS.

The FTIR graph (Fig. 9) shows spectral data for the bare carbon veil electrode and the electrode after operation in MFC-CV and MFC-CV + CS. The bending vibrations in bare carbon veil observed between regions 500–800 cm^−1^ are possibly from C–H (aromatics) or skeletal modes of aromatic rings. These peaks declined in electrodes from MFC-CV and MFC-CV + CS, suggesting breakdown or modification of aromatic structures. This suggests the common characteristics in MFCs, where the complex aromatic compounds are used up by microorganisms in the degradation process, which is when electrons are released and utilised in producing electricity. A tubular MFC was used to study the removal of benzene and toluene from groundwater, along with power generation.^23^ In total, 100% removal efficiency of benzene and toluene was observed in the MFC within 6 days of operation. This study, along with many other studies in the past, has shown the efficient removal of aromatic compounds in MFCs. Clear peaks at 1000–1200 cm^−1^ indicate C–O bonds in the carbon veil electrode. This peak was almost diminished after the operation, suggesting that C–O containing compounds such as carbonates and bicarbonates may be converted into CO_2_, volatile fatty acids, or alcohols, indicating effective substrate utilisation and transformation. Characteristic peaks related to C=C stretching around 1600 cm^−1^ and minor peaks for carbonyl (C=O) groups can be seen in bare carbon veil electrodes. This peak declined well in the electrode from MFC-CV and was found least in MFC-CV + CS, suggesting that cleavage or hydrogenation of these aromatic compounds or unsaturated structures is being broken down or modified by microbial activity. A strong peak at 2300–2400 cm^−1^ is also observed in bare carbon electrodes, which can be attributed to CO_2_ asymmetric stretching vibrations. CO_2_ from the air naturally adsorbs onto porous carbon materials, and the atmospheric CO_2_ can dissolve into moisture on the carbon surface, forming carbonate (CO_3_^2−^) and bicarbonate (HCO_3_^−^) species. Therefore, major peaks of CO_2_, carbonates and bicarbonates are seen on bare carbon electrode. A major decrease in these peaks in the carbon veil electrode from MFC-CV and MFC-CV + CS suggests possible microbial uptake and electrocatalytic conversion. The decrease in these peaks from electrodes of MFC-CV and MFC-CV + CS may also suggest loss or transformation of hydroxyl-containing compounds, possibly *via* oxidation or dehydration reactions in MFC operation.

## 5 Carbon veil integrity after 6 months of operation

**Fig. 10:**
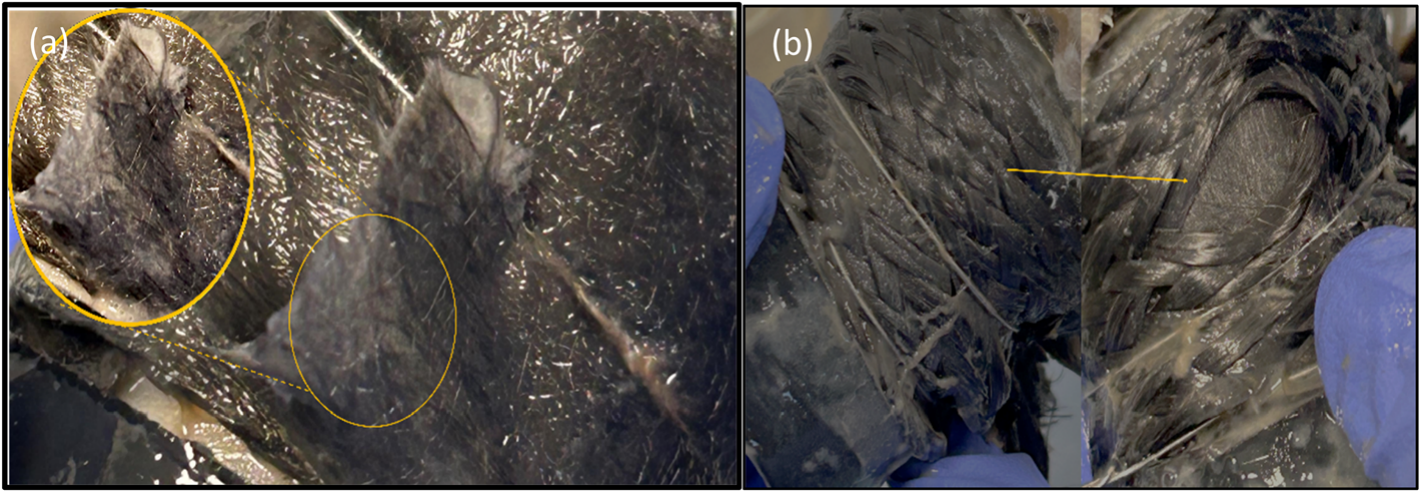
Carbon veil integrity after operation for 6 months from (a) MFC-CV with inset showing physical tear and (b) MFC-CV + CS with the arrow showing material integrity.

After 6 months of operation, electrodes were observed in both MFCs (MFC-CV and MFC-CV + CS) to compare the carbon veil integrity. The carbon veil has shown tearing after operation (Fig. 10a), which is quite expected due to its paper-like texture, whereas carbon veil wrapped inside the carbon sleeve seems to be quite intact with non-observable tearing (Fig. 10b).

## 6 Conclusions

When the carbon veil and carbon sleeve were used together, the combined electrode resulted in giving greater strength than when employed separately; this appears to be a cumulative effect, where the combination performs better than the sum of the individual components. On the other hand, the polarisation results also demonstrated that the power generation was enhanced when using a carbon sleeve in conjunction with a carbon veil. Compared to using carbon veil alone, the addition of a carbon sleeve is effective in quickly using the substrate and achieving the maximum power in a shorter time. The limitation of the carbon veil to rupture can be avoided by using a carbon sleeve over it, which will not only enhance the mechanical strength but also the electricity generation. The main difference between the two electrodes is the woven (sleeve) *vs.* non-woven (veil) makeup, which significantly affects internal resistance in the weave and weft directions. Surface area may be equally available on both electrodes, but electron flow will be subject to the local and global internal resistance of the substratum. Because of the combination of the two materials, optical imaging techniques will allow better determination of the contribution of each material separately; nevertheless, the two materials have been tested on their own, and at least MFC performance has been recorded for comparative purposes (SI). The electrode combination can be used to scale up MFCs by stacking multiple units vertically or horizontally, which can run for years. Carbon sleeve is a woven carbon fibre, whereas carbon veil is a non-woven fibre, ideal for microbial attachment, but with low tensile strength. The carbon sleeve acts as both a microbial attachment site and a mechanical reinforcement of the carbon veil. This combination seems highly suitable to scale up MFCs that can be used over a longer duration, which involves the future scope of this study.

## Author contributions

Aradhana Singh: methodology, data curation, investigation, analysis and interpretation, writing – original draft, writing – review & editing. Yi Wang: analysis and interpretation writing – review & editing. Ioannis A. Ieropoulos: conceptualization, methodology, investigation, validation, supervision, funding acquisition, writing – review & editing.

## Conflicts of interest

The authors declare that they have no known competing financial interests or personal relationships that could have appeared to influence the work reported in this paper.

## Supplementary Material

RA-015-D5RA05180K-s001

## Data Availability

The datasets generated during and/or analysed during the current study are not publicly available due to confidentiality of the results but are available from the authors upon reasonable request. Supplementary information is available. See DOI: https://doi.org/10.1039/d5ra05180k.

## References

[cit1] Ieropoulos I. A., Ledezma P., Stinchcombe A., Papaharalabos G., Melhuish C., Greenman J. (2013). Waste to real energy: The first MFC powered mobile phone. Phys. Chem. Chem. Phys..

[cit2] Naha A., Debroy R., Sharma D., Shah M. P., Nath S. (2023). Microbial fuel cell: A state-of-the-art and revolutionizing technology for efficient energy recovery. Clean. Circ. Bioeconomy.

[cit3] Kalathil S. , Patil S. A. and Pant D., Microbial fuel cells: Electrode materials, In. Encyclopedia of Interfacial Chemistry: Surface Science and Electrochemistry, Elsevier, 2018, p. 309–318

[cit4] Agrahari R., Bayar B., Abubackar H. N., Giri B. S., Rene E. R., Rani R. (2022). Advances in the development of electrode materials for improving the reactor kinetics in microbial fuel cells. Chemosphere.

[cit5] Fan Y., Hu H., Liu H. (2007). Enhanced Coulombic efficiency and power density of air-cathode microbial fuel cells with an improved cell configuration. J. Power Sources.

[cit6] Singh A., Rao A., Kaushik A. (2024). Enhancing microbial fuel cell performance for distillery wastewater treatment and bioelectricity generation: harnessing niacin as a redox mediator. Environ. Sci. Pollut. Res..

[cit7] Li S., Cheng C., Thomas A. (2017). Carbon-Based Microbial-Fuel-Cell Electrodes: From Conductive Supports to Active Catalysts. Adv. Mater..

[cit8] Merga T., Gebreslassie G., Hailu T., Nwanya A. C., Ezema F. I., Ejikeme P. M. (2025). *et al.*, Progress of carbon-based electrodes in microbial fuel cells: A comprehensive review. Results Chem..

[cit9] Ieropoulos I., Greenman J., Melhuish C. (2008). Microbial fuel cells based on carbon veil electrodes: Stack configuration and scalability. Int. J. Energy Res..

[cit10] Gajda I., Greenman J., Ieropoulos I. (2020). Microbial Fuel Cell stack performance enhancement through carbon veil anode modification with activated carbon powder. Appl. Energy.

[cit11] Artyushkova K., Roizman D., Santoro C., Doyle L. E., Fatima Mohidin A., Atanassov P. (2016). *et al.*, Anodic biofilms as the interphase for electroactive bacterial growth on carbon veil. Biointerphases.

[cit12] Gajda I., You J., Santoro C., Greenman J., Ieropoulos I. A. (2020). A new method for urine electrofiltration and long term power enhancement using surface modified anodes with activated carbon in ceramic microbial fuel cells. Electrochim. Acta.

[cit13] Bullen G. N. , Advanced Materials for Aerospace and Space Applications, In. Material and Process Modeling of Aerospace Composites, SAE International, 2019, p. 21–37

[cit14] Dworak M., Rudawski A., Markowski J., Blazewicz S. (2017). Dynamic mechanical properties of carbon fibre-reinforced PEEK composites in simulated body-fluid. Compos. Struct..

[cit15] Clauer M., Binder A. (2023). Investigation of permanent magnet synchronous machines with buried magnets and carbon fiber sleeve for automotive application. E I Elektrotechnik Inf..

[cit16] Taghavi M., Greenman J., Beccai L., Mattoli V., Mazzolai B., Melhuish C. (2014). *et al.*, High-Performance, Totally Flexible, Tubular Microbial Fuel Cell. ChemElectroChem..

[cit17] Gajda I., Greenman J., Melhuish C., Ieropoulos I. (2015). Simultaneous electricity generation and microbially-assisted electrosynthesis in ceramic MFCs. Bioelectrochemistry.

[cit18] Santoro C., Guilizzoni M., Correa Baena J. P., Pasaogullari U., Casalegno A., Li B. (2014). *et al.*, The effects of carbon electrode surface properties on bacteria attachment and start
up time of microbial fuel cells. Carbon N Y.

[cit19] Koók L., Nemestóthy N., Bélafi-Bakó K., Bakonyi P. (2021). The influential role of external electrical load in microbial fuel cells and related improvement strategies: A review. Bioelectrochemistry.

[cit20] Lee Y. S., An J., Kim B., Park H. J., Kim J., Chang I. S. (2015). Increased power in sediment microbial fuel cell: Facilitated mass transfer *via* a water-layer anode embedded in sediment. PLoS One.

[cit21] Saheb-Alam S., Persson F., Wilén B. M., Hermansson M., Modin O. (2019). Response to starvation and microbial community composition in microbial fuel cells enriched on different electron donors. Microb. Biotechnol..

[cit22] Ruiz Y., Ribot-Llobet E., Baeza J. A., Guisasola A. (2015). Conditions for high resistance to starvation periods in bioelectrochemical systems. Bioelectrochemistry.

[cit23] Lin C. W., Zhu T. J., Lin L. C., Liu S. H. (2022). Promoting biodegradation of toluene and benzene in groundwater using microbial fuel cells with cathodic modification. J. Water Proc. Eng..

