# Supplementary material for: The combined effect of a carbon sleeve and carbon veil as an anode electrode in enhancing microbial fuel cell performance
Source: RSC Adv. 2025 Nov 3;15(49):42113–20. doi: 10.1039/d5ra05180k (PMC12580964; doi:10.1039/d5ra05180k)
Supplement: RA-015-D5RA05180K-s001 [file RA-015-D5RA05180K-s001.pdf]

This journal is © The Royal Society of Chemistry 2025

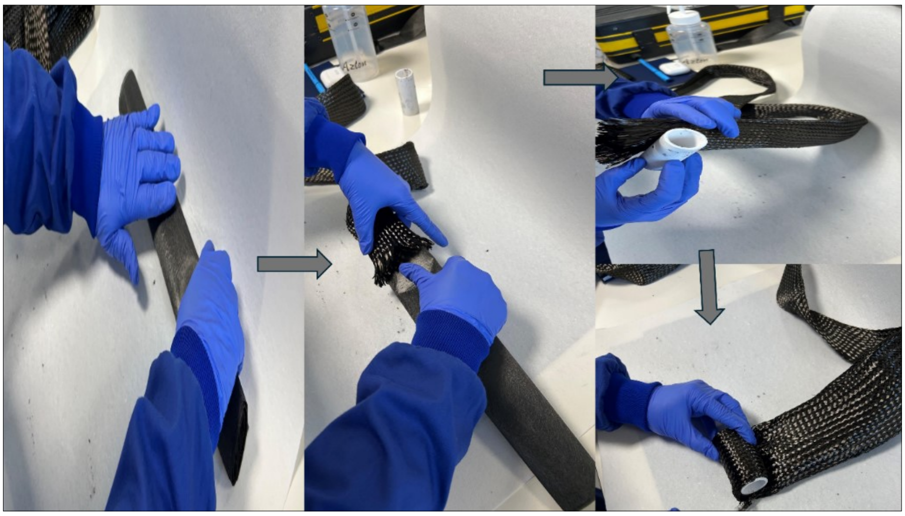

Fig S1 Placing a carbon veil inside a carbon sleeve and preparing the combination as one electrode system

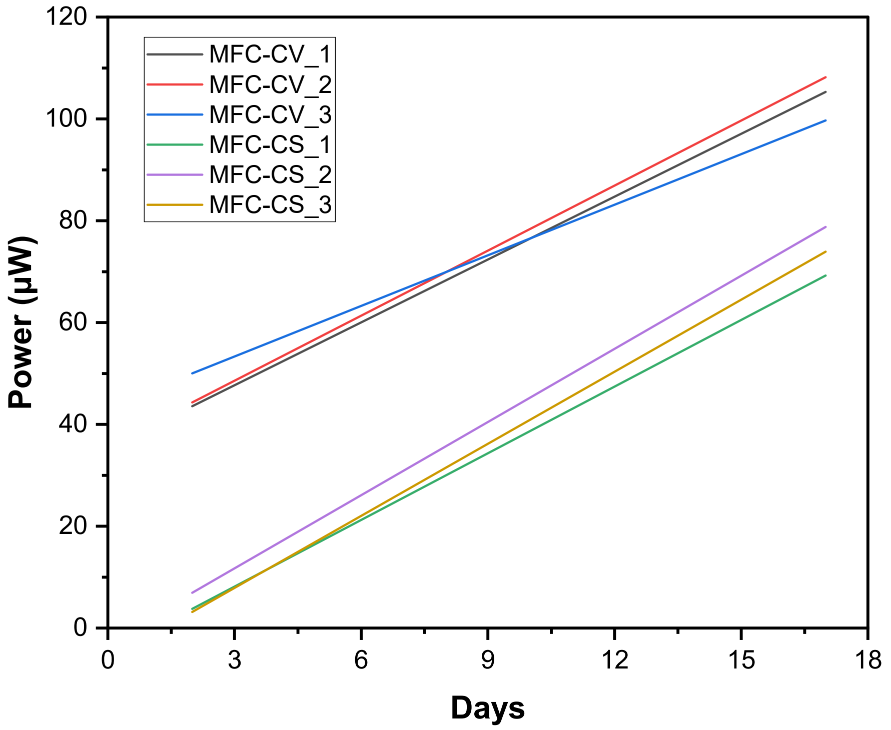

Fig. S2 Performance comparison of MFCs with carbon veil as anode electrode (MFC-CV) and MFCs with carbon sleeve as anode electrode (MFC-CS)

Absorption test in single and combination electrode

Evaluation of the combination electrode's absorption capacity of the substrate was done, and performance was compared to that of a single electrode (carbon veil). A tiny piece of a combination electrode (carbon veil + carbon sleeve) and an individual electrode (carbon veil), with similar surface area, were placed in a separate petri plate with a similar volume of feedstock. For three hours, both petri plates stayed in that position (Fig.S3). The quantity of volume absorbed on these electrodes is determined by comparing the feedstock volume before and after 3 h. A 10 % decrease in volume was observed in the combination electrode which was only 7.5 % in case of individual electrode. This demonstrates that although both electrodes have similar surface area, the volume they will absorb depends more on how carbon fibres are exposed, which will be directly proportional to the macro and micro pore structures on the electrode.

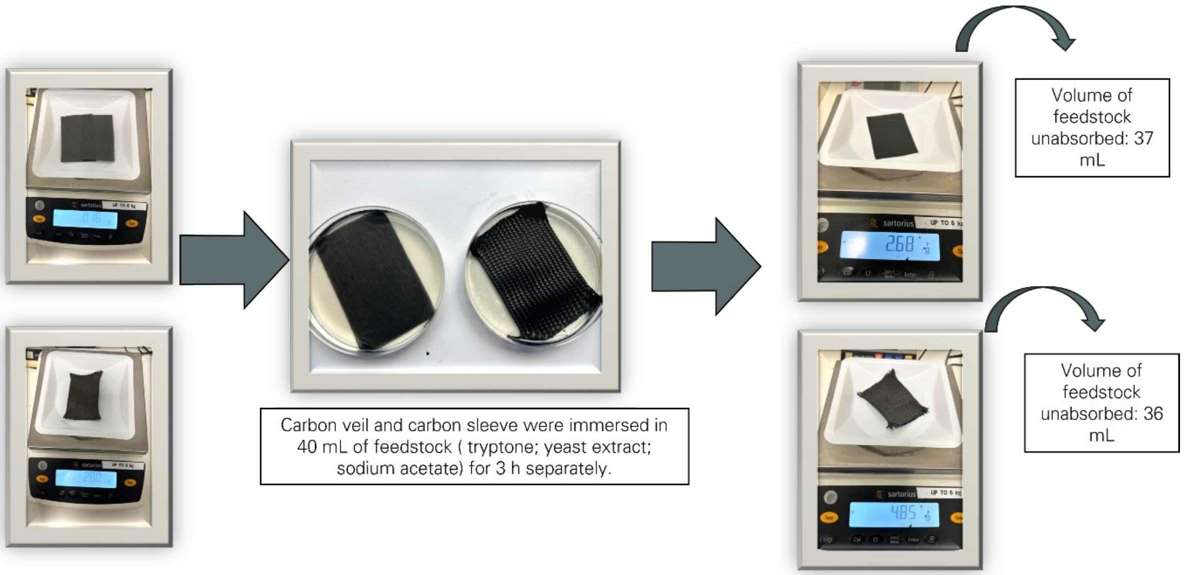

Fig.S3 Absorption test in carbon veil and combination (carbon veil + carbon sleeve) electrode
